# Data for the synthesis of new 4-aryloxy-N-arylanilines as potent succinate-cytochrome c reductase inhibitors

**DOI:** 10.1016/j.dib.2018.10.059

**Published:** 2018-10-24

**Authors:** Hua Cheng, Wei Song, Ren Nie, Yu-Xia Wang, Hui-Lian Li, Xiang-Sheng Jiang, Jun-Jun Wu, Cheng Chen, Qiong-You Wu

**Affiliations:** a Department of Chemical Engineering and Food Science, Hubei University of Arts and Science, Xiangyang 441053, PR China; b State Key Laboratory of Advanced Technology for Materials Synthesis and Processing, Wuhan University of Technology, Wuhan 430070, PR China; c Key Laboratory of Pesticide & Chemical Biology, Ministry of Education, College of Chemistry, Central China Normal University, Wuhan 430079, PR China

## Abstract

In this data article, we have designed a simple and facile protocol for copper-mediated synthesis of new 4-aryloxy-N-arylanilines under mild reaction conditions. The general information and synthetic procedures of all the target compounds were provided, and they were fully characterized by Nuclear Magnetic Resonance (NMR, including ^1^H NMR and ^13^C NMR), melting point measurements, and High-Resolution Mass Spectroscopy (HRMS). Furthermore, the inhibitory activities of these compounds against succinate-cytochrome c reductase (SCR) were evaluated, and the methods and procedures of enzyme inhibition experiments were also recorded in this data article. This article is related to “Synthesis of new 4-aryloxy-N-arylanilines and their inhibitory activities against succinate-cytochrome c reductase” (Cheng et al., 2018) [1].

## Specifications table

**Table:** 

Subject area	*Chemistry*
More specific subject area	*Organic synthesis and drug discovery.*
Type of data	*Figure*
How data was acquired	*A Bruker Avance 500 spectrometer NMR instrument,* a Buchi B-545 melting point apparatus, an Agilent 6520 Accurate-Mass Q-TOF *mass spectrometry* instrument, a Bruker DaltonicsmicroTOF-QII *mass spectrometry* instrument.
Data format	*Raw, analyzed.*
Experimental factors	*Ordinary reagents and solvents were commercially available and treated with standard methods before use.*
Experimental features	*NMR analysis: Bruker Avance 500 spectrometer NMR instrument; melting point measurements: Buchi B-545 melting point apparatus; HRMS analysis: An Agilent 6520 Accurate-Mass Q-TOF mass spectrometry instrument or a Bruker DaltonicsmicroTOF-QII mass spectrometry instrument.*
Data source location	*Xiangyang and Wuhan, China.*
Data accessibility	*Data is provided within the article.*
Related research article	*Cheng H, Song W, Nie R, Wang YX, Li HL, Jiang XS, Wu JJ, Chen C, Wu QY. Synthesis of new 4-aryloxy-N-arylanilines and their inhibitory activities against succinate-cytochrome c reductase. Bioorg Med Chem Lett. 2018 28:1330-1335.*[1]

## Value of the data

• Data presented here provided the general information and synthetic procedures of various new organic molecules, which will be a valuable guidance for organic chemists.
• Data presented here contained full characterization of various new compounds, which will be useful for further scientists to confirm their structures if they are interested in the synthesis of the compounds in this data article.
• Data presented here included a detailed explanation about how enzyme inhibition assays were conducted, which could guild those who aim to do similar experiments.

## 1 Data

**Fig. 1:**
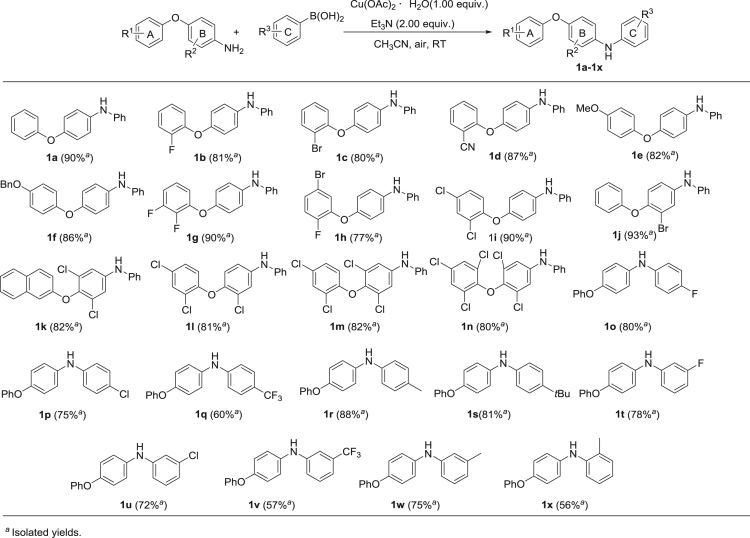
Synthesis of compounds **1a**–**1x**.

A new series of 4-aryloxy-N-arylanilines (**1a**–**1x**) were synthesized by a copper-mediated strategy, and the synthetic scheme as well as the structures of all target compounds were depicted in Fig. 1. Besides, the inhibitory activities of **1a**–**1x** against succinate-cytochrome c reductase (SCR, a mixture of mitochondrial complex II and complex III) were tested and some compounds demonstrated attractive performance. The methods and procedures for the enzyme inhibition assays were provided in this data article.

## 2 Experimental design, materials, and methods

### 2.1 General considerations

^1^H NMR (^13^C NMR) spectra were recorded on a Bruker Avance 500 (126 MHz) spectrometer in CDCl_3_ with TMS as the internal reference. HRMS was analyzed on an Agilent 6520 Accurate-Mass Q-TOF or a Bruker Daltonics microTOF-QII instrument, and melting points were taken on a Buchi B-545 melting point apparatus. The copper salts, solvents, boronic acids and some anilines were purchased from commercial suppliers. Moreover, most of the anilines were prepared according to a literature report [2].

### 2.2 General procedure for synthesis of compounds 1a–1x

Synthesis of **1a**–**1x** followed a classic synthetic procedure for the Chan-Lam coupling of a boronic acid and an amine aided by a copper salt, and the pure products were obtained by column chromatography. See [1] and Supporting information of [1] for the detailed procedure.

### 2.3 Enzyme assay

The overall activities of SCR, complex II and complex III were determined using our previous procedures [3], [4]. The preparation of SCR from the porcine heart was essential as reported [5], and DBH_2_ was prepared from DB according to the procedure described in a previous publication [3]. In addition, the absolute IC_50_ values for all experiments were obtained from a reported method [4]. See [1] and Supporting information of [1] for the detailed procedure.

### 2.4 Characterization data

All the NMR and HRMS data for the target products are supplied in Supplementary information.

## Figures and Tables

**Fig. S1:**
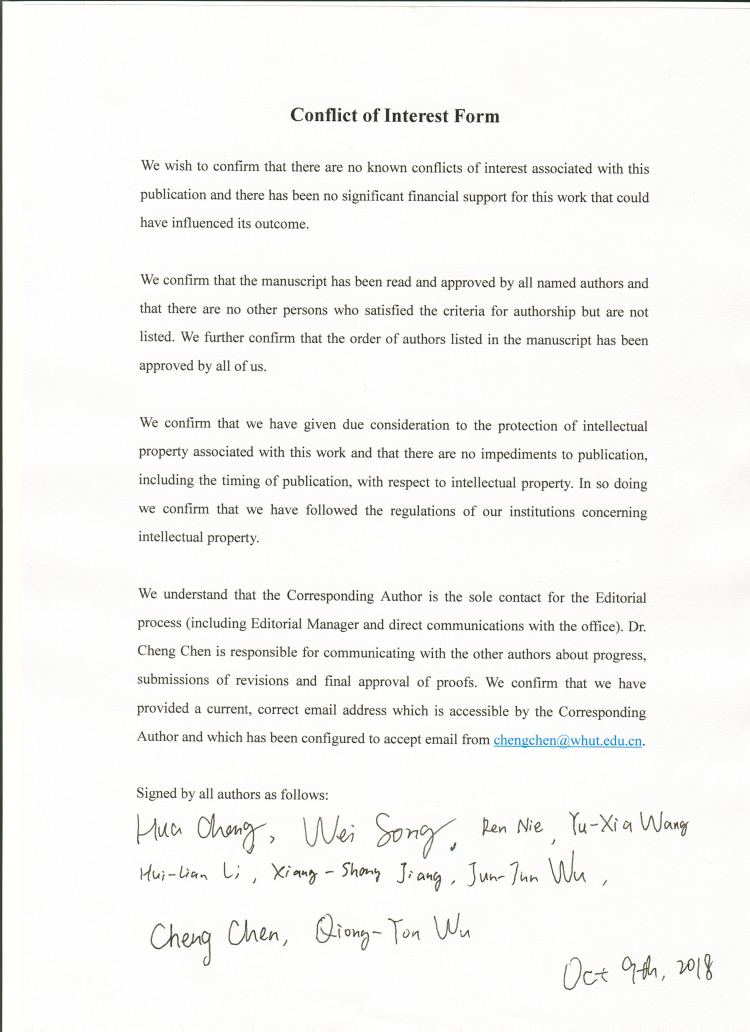


## References

[1] Cheng H., Song W., Nie R., Wang Y.X., Li H.L., Jiang X.S., Wu J.J., Chen C., Wu Q.Y. (2018). Synthesis of new 4-aryloxy-N-arylanilines and their inhibitory activities against succinate-cytochrome c reductase. Bioorg. Med. Chem. Lett..

[2] Cheng H., Shen Y.Q., Pan X.Y., Hou Y.P., Wu Q.Y., Yang G.F. (2015). Discovery of 1,2,4-triazole-1,3-disulfonamides as dual inhibitors of mitochondrial complex II and complex III. New J. Chem..

[3] Zhu X.L., Xiong L., Li H., Song X.Y., Liu J.J., Yang G.F. (2014). Computational and experimental insight into the molecular mechanism of carboxamide inhibitors of succinate-ubquinone oxidoreductase. ChemMedChem.

[4] Xiong L., Zhu X.L., Shen Y.Q., Wishwa W.K.W.M., Li K., Yang G.F. (2015). Discovery of N-benzoxazol-5-yl-pyrazole-4-carboxamides as nanomolar SQR inhibitors. Eur. J. Med. Chem..

[5] Yu L., Yu C.A. (1982). Quantitative resolution of succinate-cytochrome c reductase into succinate-ubiquinone and ubiquinol-cytochrome c reductases. J. Biol. Chem..

