# Supplementary material for: Data for the synthesis of new 4-aryloxy-N-arylanilines as potent succinate-cytochrome c reductase inhibitors
Source: Data Brief. 2018 Oct 24;21:878–81. doi: 10.1016/j.dib.2018.10.059 (PMC6223225; doi:10.1016/j.dib.2018.10.059)
Supplement: Supplementary file 1 — Supplementary material [file mmc2.docx]

**Supporting Information for Data Article**

**Title**: *Data for the synthesis of new 4-aryloxy-N-arylanilines as potent succinate-cytochrome c reductase inhibitors*

**Authors**: Hua Cheng ^a^, Wei Song ^a^, Ren Nie ^a^, Yu-Xia Wang ^c^, Hui-Lian Li ^a^, Xiang-Sheng Jiang ^a^, Jun-Jun Wu ^a^, Cheng Chen^b,^* and Qiong-You Wu ^c,^*

**Affiliations**:

*^a^ Department of Chemical Engineering and Food Science, Hubei University of Arts and Science, Xiangyang 441053, P. R. China;*

*^b^ State Key Laboratory of Advanced Technology for Materials Synthesis and Processing, Wuhan University of Technology, Wuhan 430070, P. R. China.*

*^c^ Key Laboratory of Pesticide & Chemical Biology, Ministry of Education, College of Chemistry, Central China Normal University, Wuhan 430079, P. R. China.*

**Contact emails**: [chengchen@whut.edu.cn](mailto:chengchen@whut.edu.cn); qywu@mail.ccnu.edu.cn

**Characterization data for compounds 1a-1x**

*4-Phenoxy-N-phenylaniline (****1a****)*. White solid: m.p. 99.5-101.0 ^o^C. Isolated yield: 90%. ^1^H NMR (500 MHz, CDCl_3_) δ 7.45 – 7.12 (m, 4H), 7.10-6.80 (m, 10H), 5.52 (brs, 1H). ^13^C NMR (126 MHz, CDCl_3_) δ 158.13, 151.17, 143.89, 138.72, 129.61, 129.33, 122.60, 120.46, 120.33, 117.90, 116.83. HRMS (ESI): m/z calcd. for C_18_H_16_NO [M+H]^+^: 262.12264; Found: 262.12257.

*4-(2-Fluorophenoxy)-N-phenylaniline (****1b****)*. White solid: m.p. 81.9-82.5 ^o^C. Isolated yield: 81%. ^1^H NMR (500 MHz, CDCl_3_) δ 7.35 – 7.30 (m, 2H), 7.26 – 7.21 (m, 1H), 7.16 – 6.94 (m, 11H), 5.64 (s, 1H). ^13^C NMR (126 MHz, CDCl_3_) δ 154.91, 152.94, 151.45, 144.83, 144.75, 143.88, 138.63, 129.31, 124.51, 124.48, 124.01, 123.96, 120.66, 120.41, 120.34, 118.97, 116.96, 116.82, 116.76. HRMS (ESI): m/z calcd. for C_18_H_15_FNO [M+H]^+^: 280.11322; Found: 280.11325.

*4-(2-Bromophenoxy)-N-phenylaniline (****1c****).* Yellow solid: m.p. 76.0-76.6 ^o^C. Isolated yield: 80%. ^1^H NMR (500 MHz, CDCl_3_) δ 7.60 (d, *J* = 7.6 Hz, 1H), 7.26 – 7.17 (m, 3H), 7.05 (d, *J* = 8.2 Hz, 2H), 7.02 – 6.84 (m, 7H), 5.59 (s, 1H). ^13^C NMR (126 MHz, CDCl_3_) δ 154.59, 150.84, 143.75, 138.97, 133.69, 129.34, 128.50, 124.24, 120.56, 120.20, 119.87, 119.19, 116.92, 113.97. HRMS (ESI): m/z calcd. for C_18_H_15_BrNO [M+H]^+^: 340.03315; Found: 340.03307.

*2-(4-(Phenylamino)phenoxy)benzonitrile (****1d****).* Pale yellow oil. Isolated yield: 87%. ^1^H NMR (500 MHz, CDCl_3_) δ 7.60 (d, *J* = 6.4 Hz, 1H), 7.48 – 7.40 (m, 1H), 7.30 – 7.22 (m, 2H), 7.06 (d, *J* = 7.2 Hz, 5H), 7.02 – 6.89 (m, 3H), 6.84 (d, *J* = 7.7 Hz, 1H), 5.79 (s, 1H). ^13^C NMR (126 MHz, CDCl_3_) δ 160.71, 148.51, 143.25, 140.67, 134.29, 133.86, 129.48, 122.35, 121.50, 121.15, 119.44, 117.68, 116.34, 116.12, 102.99. HRMS (ESI): m/z calcd. for C_19_H_15_N_2_O [M+H]^+^: 287.11789; Found: 287.11767.

*4-(4-Methoxyphenoxy)-N-phenylaniline (****1e****).* Pink solid: m.p. 83.3-84.8 ^o^C. Isolated yield: 82%. ^1^H NMR (500 MHz, CDCl_3_) δ 7.30 (d, *J* = 7.1 Hz, 2H), 7.11 (d, *J* = 7.8 Hz, 2H), 7.05 (d, *J* = 6.5 Hz, 4H), 7.02 – 6.90 (m, 5H), 5.64 (s, 1H), 3.86 (s, 3H). ^13^C NMR (126 MHz, CDCl_3_) δ 155.39, 152.71, 151.04, 144.12, 137.84, 129.23, 120.65, 120.10, 119.81, 119.06, 116.41, 114.71, 55.54. HRMS (ESI): m/z calcd. for C_19_H_18_NO_2_ [M+H]^+^: 292.13321; Found: 292.13317.

*4-(4-(Benzyloxy)phenoxy)-N-phenylaniline (****1f****).* Pale yellow oil. Isolated yield: 86%. ^1^H NMR (500 MHz, CDCl_3_) δ 7.51 (d, *J* = 7.0 Hz, 2H), 7.46 (dd, *J* = 7.1 Hz, 2H), 7.43 – 7.38 (m, 1H), 7.31 (dd, *J* = 7.2 Hz, 2H), 7.11 (d, *J* = 8.1 Hz, 2H), 7.08 – 6.92 (m, 9H), 5.63 (s, 1H), 5.11 (s, 2H). ^13^C NMR (126 MHz, CDCl_3_) δ 154.61, 152.60, 151.38, 144.13, 137.97, 136.99, 129.28, 128.52, 127.91, 127.43, 120.65, 120.18, 119.74, 119.25, 116.49, 115.83, 70.49. HRMS (ESI): m/z calcd. for C_25_H_22_NO_2_ [M+H]^+^: 368.16451; Found: 368.16449.

*4-(2,3-Difluorophenoxy)-N-phenylaniline (****1g****).* White solid: m.p. 70.7-72.2 ^o^C. Isolated yield: 90%. ^1^H NMR (500 MHz, CDCl_3_) δ 7.41 – 7.27 (m, 2H), 7.19 – 6.88 (m, 9H), 6.85 – 6.74 (m, 1H), 5.66 (s, 1H). ^13^C NMR (126 MHz, CDCl_3_) δ 152.64, 152.56, 150.67, 150.59, 150.49, 146.92, 143.66, 143.56, 141.67, 141.56, 139.39, 139.35, 129.37, 129.34, 123.29, 120.73, 120.71, 119.94, 119.64, 119.61, 117.12, 117.09, 114.81, 111.38, 111.36, 111.24, 111.22. HRMS (ESI): m/z calcd. for C_18_H_14_F_2_NO [M+H]^+^: 298.10380; Found: 298.10380.

*4-(5-Bromo-2-fluorophenoxy)-N-phenylaniline (****1h****).* White solid: m.p. 55.9-57.4 ^o^C. Isolated yield: 77%. ^1^H NMR (500 MHz, CDCl_3_) δ 7.30 (dd, *J* = 7.2, 7.0 Hz, 2H), 7.18 (d, *J* = 6.4 Hz, 1H), 7.13 (d, *J* = 7.4 Hz, 1H), 7.10 (d, *J* = 8.0 Hz, 2H), 7.06 (d, *J* = 7.4 Hz, 3H), 7.02 – 6.93 (m, 3H), 5.66 (s, 1H). ^13^C NMR (126 MHz, CDCl_3_) δ 153.74, 151.76, 149.99, 146.25, 146.15, 143.40, 139.58, 129.34, 126.32, 126.27, 122.72, 122.71, 120.81, 119.81, 119.75, 118.18, 118.02, 117.25, 116.25, 116.23. HRMS (ESI): m/z calcd. forC_18_H_14_BrFNO [M+H]^+^: 358.02373; Found: 358.02358.

*4-(2,4-Dichlorophenoxy)-N-phenylaniline (****1i****)*. Yellow solid: m.p. 64.1-65.0 ^o^C. Isolated yield: 90%. ^1^H NMR (500 MHz, CDCl_3_)δ 7.49 (s, 1H), 7.31 (dd, *J* = 7.4, 7.2 Hz, 2H), 7.19 (d, *J* = 8.7 Hz, 1H), 7.10 (d, *J* = 8.1 Hz, 2H), 7.06 (d, *J* = 7.6 Hz, 2H), 6.99 – 6.94 (m, 3H), 6.90 (d, *J* = 8.7 Hz, 1H), 5.66 (s, 1H). ^13^C NMR (126 MHz, CDCl_3_) δ 152.37, 150.27, 143.46, 139.31, 130.24, 129.32, 128.15, 127.78, 125.50, 120.71, 119.90, 119.77, 117.08.HRMS (ESI): m/z calcd. for C_18_H_14_Cl_2_NO [M+H]^+^: 330.04470; Found: 330.04468.

*3-Bromo-4-phenoxy-N-phenylaniline (****1j****).* Yellow oil. Isolated yield: 93%. ^1^H NMR (500 MHz, CDCl_3_) δ 7.42 – 7.28 (m, 5H), 7.15 – 7.07 (m, 3H), 7.05 – 6.90 (m, 5H), 5.68 (s, 1H). ^13^C NMR (126 MHz, CDCl_3_) δ 157.79, 146.75, 142.52, 140.72, 129.60, 129.43, 122.58, 122.38, 122.29, 121.56, 118.09, 118.03, 116.87, 116.15. HRMS (ESI): m/z calcd. for C_18_H_15_BrNO [M+H]^+^: 340.03315; Found: 340.03236.

*3,5-Dichloro-4-(naphthalen-2-yloxy)-N-phenylaniline (****1k****)*. White solid: m. p. 95.6-96.9 ^o^C. Isolated yield: 82%. ^1^H NMR (500 MHz, CDCl_3_) δ 8.55 (s, 1H), 7.89 (s, 1H), 7.59 (s, 3H), 7.38 – 7.30 (m, 3H), 7.20 – 7.03 (m, 5H), 6.54 (s, 1H), 5.71 (s, 1H). ^13^C NMR (126 MHz, CDCl_3_) δ 152.98, 142.06, 141.23, 140.44, 134.79, 130.27, 129.68, 127.53, 126.63, 125.69, 125.44, 124.90, 122.96, 122.11, 122.08, 119.72, 116.60, 106.67. HRMS (ESI): m/z calcd. forC_22_H_16_Cl_2_NO [M+H]^+^: 380.06035; Found: 380.05977.

*3-Chloro-4-(2,4-dichlorophenoxy)-N-phenylaniline*(**1l**). White solid: m.p. 70.0-71.5 ^o^C. Isolated yield: 81%. ^1^H NMR (500 MHz, CDCl_3_) δ 7.48 (d, *J* = 2.4 Hz, 1H), 7.33 (dd, *J* = 7.8, 7.6 Hz, 2H), 7.19 (d, *J* = 1.6 Hz, 1H), 7.15 (dd, *J* = 8.8, 2.4 Hz, 1H), 7.10 (d, *J* = 7.9 Hz, 2H), 7.03 (dd, *J* = 7.6, 7.4 Hz, 1H), 6.95 (d, *J* = 1.4 Hz, 2H), 6.71 (d, *J* = 8.8 Hz, 1H), 5.71 (s, 1H). ^13^C NMR (126 MHz, CDCl_3_) δ 152.28, 144.68, 142.19, 141.41, 130.39, 129.57, 128.04, 127.74, 126.83, 124.57, 122.26, 122.07, 119.05, 118.59, 117.95, 116.99. HRMS (APCI): m/z calcd. for C_18_H_13_Cl_3_NO [M+H]^+^: 364.00572; Found: 364.00558.

*3,5-Dichloro-4-(2,4-dichlorophenoxy)-N-phenylaniline* (**1m**). White solid: m.p. 111.2-112.7 ^o^C. Isolated yield: 82%. ^1^H NMR (500 MHz, CDCl_3_) δ 7.48 (d, *J* = 2.4 Hz, 1H), 7.38 (dd, *J* = 7.8, 7.6 Hz, 2H), 7.19 – 7.08 (m, 4H), 7.04 (s, 2H), 6.50 (d, *J* = 8.8 Hz, 1H), 5.77 (s, 1H). ^13^C NMR (126 MHz, CDCl_3_) δ 151.63, 142.71, 140.81, 139.42, 130.41, 129.93, 129.73, 127.51, 123.36, 123.33, 120.15, 116.14, 115.04. HRMS (APCI): m/z calcd. for C_18_H_12_Cl_4_NO [M+H]^+^: 397.96675; Found: 397.96847.

*3,5-Dichloro-N-phenyl-4-(2,4,6-trichlorophenoxy)aniline* (**1n**). White solid: m.p. 103.4-104.8 ^o^C. Isolated yield: 80%. ^1^H NMR (500 MHz, CDCl_3_) δ 7.39 – 7.31 (m, 4H), 7.11 (d, *J* = 7.9 Hz, 2H), 7.07 (dd, *J* = 7.6, 7.4 Hz, 1H), 6.99 (s, 2H), 5.63 (brs, 1H). ^13^C NMR (126 MHz, CDCl_3_) δ 147.79, 141.94, 141.27, 140.77, 129.67, 129.10, 128.89, 127.36, 127.06, 122.87, 119.44, 117.04. HRMS (APCI): m/z calcd. for C_18_H_11_Cl_5_NO [M+H]^+^: 431.92778; Found: 431.92599.

*4-Fluoro-N-(4-phenoxyphenyl)aniline (****1o****)*. White solid: m.p. 71.1-72.5 ^o^C. Isolated yield: 80%. ^1^H NMR (500 MHz, CDCl_3_) δ 7.35 (dd, *J* = 7.4, 7.2 Hz, 2H), 7.18 – 7.08 (m, 2H), 7.07 – 6.94 (m, 9H), 5.53 (brs, 1H). ^13^C NMR (126 MHz, CDCl_3_) δ 158.66, 158.21, 156.76, 150.83, 139.77, 139.75, 139.62, 129.92, 129.62, 124.47, 124.06, 122.58, 120.62, 119.65, 119.43, 119.37, 119.24, 118.42, 117.82, 115.99, 115.81. HRMS (ESI): m/z calcd. for C_18_H_15_FNO [M+H]^+^: 280.11322; Found: 280.11285.

*4-Chloro-N-(4-phenoxyphenyl)aniline (****1p****)*. White solid: m.p. 80.0-81.2 ^o^C. Isolated yield: 75%. ^1^H NMR (500 MHz, CDCl_3_) δ 7.31 (dd, *J* = 7.6, 7.4 Hz, 2H), 7.18 (d, *J* = 8.1 Hz, 2H), 7.09 – 6.86 (m, 9H), 5.64 (brs, 1H). ^13^C NMR (126 MHz, CDCl_3_) δ 157.98, 151.82, 142.69, 138.15, 129.71, 129.31, 125.05, 122.84, 120.84, 120.48, 118.12, 117.89. HRMS (ESI): m/z calcd. for C_18_H_15_ClNO [M+H]^+^: 296.08367; Found: 296.08445.

*4-Phenoxy-N-(4-(trifluoromethyl)phenyl)aniline (****1q****)*. White solid: m.p. 91.0-92.5 ^o^C. Isolated yield: 60%. ^1^H NMR (500 MHz, CDCl_3_) δ 7.44 (d, *J* = 5.9 Hz, 2H), 7.33 (s, 2H), 7.21 – 6.89 (m, 9H), 5.79 (brs, 1H). ^13^C NMR (126 MHz, CDCl_3_) δ 157.60, 153.03, 147.57, 136.43, 129.75, 127.88, 126.71, 126.68, 125.73, 123.57, 123.10, 122.83, 121.56, 121.42, 121.30, 121.04, 120.78, 120.22, 118.41, 114.52. HRMS (ESI): m/z calcd. for C_19_H_15_F_3_NO [M+H]^+^: 330.11003; Found: 330.10990.

*4-Methyl-N-(4-phenoxyphenyl)aniline (****1r****)*. White solid: m.p. 69.0-70.5 ^o^C. Isolated yield: 88%. ^1^H NMR (500 MHz, CDCl_3_) δ 7.30 (dd, *J* = 7.6 Hz, 2H), 7.13 – 6.82 (m, 11H), 5.51 (brs, 1H), 2.29 (s, 3H). ^13^C NMR (126 MHz, CDCl_3_) δ 158.35, 150.54, 141.07, 139.74, 130.42, 129.87, 129.60, 122.47, 120.62, 119.20, 117.95, 117.76, 20.62. HRMS (ESI): m/z calcd. for C_19_H_18_NO [M+H]^+^: 276.13829; Found: 276.13500.

*4-(Tert-butyl)-N-(4-phenoxyphenyl)aniline (****1s****)*. Pale yellow oil. Isolated yield: 81%. ^1^H NMR (500 MHz, CDCl_3_) δ 7.35 – 7.24 (m, 4H), 7.08 – 7.00 (m, 3H), 7.00 – 6.89 (m, 6H), 1.30 (s, 9H). ^13^C NMR (126 MHz, CDCl_3_) δ 158.36, 150.73, 143.81, 141.14, 139.50, 129.65, 126.18, 122.54, 120.62, 119.59, 117.84, 117.28, 31.51, 29.74. HRMS (ESI): m/z calcd. for C_22_H_24_NO [M+H]^+^: 318.18524; Found: 318.18550.

*3-Fluoro-N-(4-phenoxyphenyl)aniline (****1t****).* White solid: m.p. 69.5-71.0 ^o^C. Isolated yield: 78%. ^1^H NMR (500 MHz, CDCl_3_) δ 7.39 (d, *J* = 3.4 Hz, 2H), 7.25 – 7.19 (m, 1H), 7.16 – 7.11 (m, 3H), 7.10 – 7.02 (m, 4H), 6.74 (d, *J* = 10.6 Hz, 2H), 6.61 (s, 1H), 5.64 (s, 1H). ^13^C NMR (126 MHz, CDCl_3_) δ 164.78, 162.84, 157.80, 157.79, 152.17, 146.22, 146.13, 137.37, 137.36, 130.48, 130.40, 129.68, 129.67, 122.86, 122.86, 121.72, 120.29, 118.16, 111.61, 106.53, 106.36, 102.71, 102.51. HRMS (ESI): m/z calcd. for C_18_H_15_FNO [M+H]^+^: 280.11322; Found: 280.11320.

*3-Chloro-N-(4-phenoxyphenyl)aniline* (***1u***). Yellow oil. Isolated yield: 72%. ^1^H NMR (500 MHz, CDCl_3_) δ 7.36 (dd, *J* = 7.6 Hz, 2H), 7.19 – 7.08 (m, 4H), 7.06 – 7.00 (m, 4H), 6.98 (s, 1H), 6.85 (dd, *J* = 7.7 Hz, 2H), 5.65 (brs, 1H). ^13^C NMR (126 MHz, CDCl_3_) δ 157.77, 152.21, 145.64, 137.32, 135.00, 130.30, 129.67, 122.87, 121.69, 120.30, 119.86, 118.19, 115.66, 114.16. HRMS (ESI): m/z calcd. for C_18_H_15_ClNO [M+H]^+^: 296.08367; Found: 296.08354.

*N-(4-phenoxyphenyl)-3-(trifluoromethyl)aniline* (***1v***). Yellow oil. Isolated yield: 57%. ^1^H NMR (500 MHz, CDCl_3_) δ 7.46-7.30 (m, 3H), 7.21 (s, 1H), 7.16 – 7.09 (m, 5H), 7.09 – 6.99 (m, 4H), 5.63 (s, 1H). ^13^C NMR (126 MHz, CDCl_3_) δ 157.72, 152.51, 144.87, 137.12, 132.12, 131.86, 131.61, 131.36, 129.81, 129.72, 127.37, 125.20, 123.03, 122.98, 121.80, 120.87, 120.33, 118.76, 118.31, 116.40, 116.37, 116.34, 116.31. HRMS (ESI): m/z calcd. for C_19_H_15_F_3_NO [M+H]^+^: 330.11003; Found: 330.11002.

*3-Methyl-N-(4-phenoxyphenyl)aniline (****1w****)*. White solid: m.p. 59.8-61.0 ^o^C. Isolated yield: 75%. ^1^H NMR (500 MHz, CDCl_3_) δ 7.39 (dd, *J* = 7.4, 7.2 Hz, 2H), 7.21 (dd, *J* = 7.8, 7.6 Hz, 1H), 7.16 – 7.10 (m, 3H), 7.08 (d, *J* = 7.8 Hz, 2H), 7.03 (d, *J* = 8.0 Hz, 2H), 6.89 (s, 2H), 6.80 (d, *J* = 7.3 Hz, 1H), 5.57 (brs, 1H), 2.38 (s, 3H). ^13^C NMR (126 MHz, CDCl_3_) δ 158.14, 151.02, 143.80, 139.16, 138.85, 129.59, 129.15, 122.56, 121.36, 120.42, 120.24, 117.88, 117.56, 113.97, 21.48. HRMS (ESI): m/z calcd. for C_19_H_18_NO [M+H]^+^: 276.13829; Found: 276.13785.

*2-Methyl-N-(4-phenoxyphenyl)aniline (****1x****)*. Pale yellow oil. Isolated yield: 56%. ^1^H NMR (500 MHz, CDCl_3_) δ 7.36 (dd, *J* = 7.7 Hz, 2H), 7.25-7.15 (m, 3H), 7.10 (dd, *J* = 7.3 Hz, 1H), 7.07-6.99 (m, 6H), 6.94 (dd, *J* = 7.2, 7.0 Hz, 1H), 5.29 (brs, 1H), 2.31 (s, 3H). ^13^C NMR (126 MHz, CDCl_3_) δ 158.25, 150.83, 142.00, 139.52, 130.88, 129.59, 127.04, 126.78, 122.52, 121.27, 120.53, 120.01, 117.82, 117.25, 17.80. HRMS (ESI): m/z calcd. for C_19_H_18_NO [M+H]^+^: 276.13829; Found: 276.13798.
